# The complete chloroplast genome of *Ixora chinensis* and phylogenetic relationships

**DOI:** 10.1080/23802359.2021.1989336

**Published:** 2021-10-15

**Authors:** Ana Bian, Luanmei Lu

**Affiliations:** Key Laboratory of Landscape Plants with Fujian and Taiwan Characteristics of Fujian Colleges and Universities, Department of Biological Science and Biotechnology, Minnan Normal University, Zhangzhou, Fujian, China

**Keywords:** *Ixora chinensis*, Rubiaceae, chloroplast genome, phylogenetic analysis, include these here if the journal requires them

## Abstract

In this study, the complete chloroplast (cp) genome of *Ixora chinensis* was sequenced by next-generation sequencing for the first time. The complete cp genome is 154,787 in length and contained 131 genes, consisting of 86 protein-coding genes, eight ribosomal RNA genes, and 37 transfer RNA genes. The phylogenetic position based on chloroplast genomes suggests that *I. chinensis* was closely related to *I. chinensis* (MN850660.1) within the Ixora clade, which may provide useful information for further understanding the evolution of *I. chinensis.*

*Ixora chinensis* Lam. also named ‘Dauk Chem’ is native to China and Malaysia, belonging to the genus of Ixora in the Rubiaceae family. In recent years, the flower has become more and more popular in China. It is widely distributed in the southwest and southeast regions of China, such as Hainan, Fujian, Guangdong and Guangxi (Li et al. 2019). *Ixora chinensis* has a longer vase life than other Ixora species which makes it an outstanding cut flower (Chen et al. 2003). In addition, most of the activities reported on leaves and flowers of *I. chinensis* were anti-oxidants, anticancer, anti-microbial activities (Bhagyasri et al. 2019). Interestingly, the flower with bright and abundant flowers has a long blooming term lasting for almost the entire year. *Ixora chinensis* with red flowers is one of the most common native species in southern China, not only with ornamental value but more importantly with its medicinal values which it is rich in flavonoids and anthocyanins (Rastogi and Mehrotra 1990). Recently, *I. chinensis* was assembled by Ly et al. (2020) to comparative genomics and molecular phylogeny in subfamily Ixoroideae. However, it was not assembled into a circular chloroplast genomes and the cp genome was not annotated. In addition, the chloroplast sequence (accession number: MN850660.1) is not verified by NCBI. There is yet little information on the complete chloroplast (cp) genome of *I. chinensis.* Here, the complete chloroplast (cp) genome of *I. chinensis* was assembled and annotated for the first time. We also carried out a systematic comparative genomic study of I chinensis plastome and those from another two Ixora species, in order to explore its phylogenetic relationship and identify new regions of genomic variability.

The plant material of fresh leaves for extraction was collected from Caiban Village, Jiuhu Town, Zhangzhou (Fujian, China, 117°59′56″E, 24°48′40″N). Total chloroplast DNA was extracted following Kim et al. (2006) with the optimized CTAB method, and used to construct to build an Illumina pair-end library. Then the library was done on Illumina Novaseq 6000 platform at Beijing Genomics Institute (BGI, Shenzhen, China) and generating approximately 3.6 G of raw data. The quality of reads was filtered using the FastQC program (Andrews 2014). High-quality clean reads were de novo assembled using SPAdes version 3.11.0 software (Bankevich et al. 2012) the cp genome was annotated using the online program GeSeq (Tillich et al. 2017) with the chloroplast genome of *Vangueria infausta* (accession number MN851269) as reference. The annotated chloroplast genome sequence of was deposited in GenBank under the accession number MZ221832. The voucher specimens of *I. chinensis* was stored at laboratory of Department of Biological Science and Biotechnology, Minnan Normal University, Zhangzhou (URL: https://bio.mnnu.edu.cn/info/1110/2223.htm, accession num-ber: No.MNU001) (Ana Bian, Email: 549511030@qq.com).

The complete length of *I. chinensis* is 154,787 bp (Figure 1), longer than *I. chinensis* (MN850660.1, length = 154,665 bp, GC = 37.48%) and overall GC content of 37.5%. The cp genome of *I. chinensis* has a typical quadripartite structure, containing a large single-copy region of 84,880 bp, a small single-copy region of 18,177 bp, and a pair of inverted repeat regions of 25,865 bp, is longer than *I. chinensis* (MN850660.1, LSC = 84,874 bp, SSC= 18,157 bp, IR = 25,817 bp). We annotated the cp genome of *I. chinensis* (MN850660.1). The results show that there is no difference in the gene contents between *I. chinensis* (MZ221832.1) and *I. chinensis* (MN850660.1). The CP genome comprised 131 genes, including 86 protein-coding genes, 37 transfer RNA genes (tRNA), and 8 ribosomal RNA genes (rRNA). The chloroplast genome of *I. chinensis* has 17 different intron containing genes, exhibiting two introns (*clp*P and *ycf*3) and the rest of the genes contained one intron (*trn*K-UUU, *rps*16, *trn*G-UCC, *atp*F, *rpo*C1, *trn*L-UAA, *trn*V-UAC, *pet*B, *pet*D, *rpl*16, *rpl*2, *ndh*B, *trn*I-GAU, *trn*A-UGC, *ndh*A).

**Figure 1.**
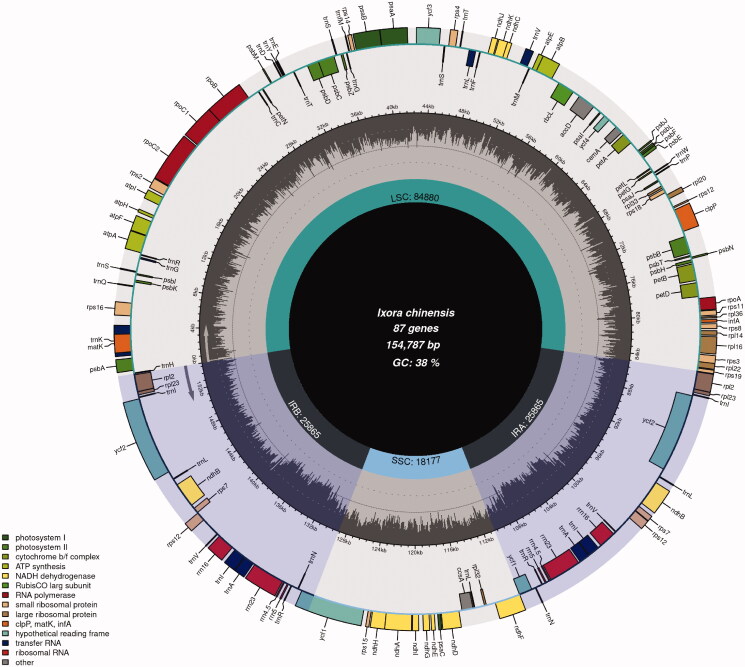
The schematic representation of the plastome of *I. chinensis* created.

To explore the ixora species’ gene gene pseudonization and deletion scale, we performed a two-way clustering analysis of 3 Ixora species and use *A. thaliana* as a reference (Figure 2). We found that the tRNA (trnA-Y) and genes from the ycf family (ycf5, ycf6, ycf9, ycf10) were lost in all Ixora plastomes compared with that of *A. thaliana* (Figure 2). Polymorphism analysis of 3 Ixora species shows that petD gene has a higher variation (Figure 3). Sequence divergence was visualized using mVISTA with *I. chinensis* (MN850660.1) as the reference annotated genome. Globally, sequence divergence among all ixora was relatively high and mainly concentrated in conserved non-coding sequences and in Untranslated Transcribed Regions (UTR). However, variation among species seemed to be negligible for UTRs located in the IR region. Substitutions were more frequent but indels (trnV-UAC) were observed as well in *I. hookeri* (Figure 4).

**Figure 2.**
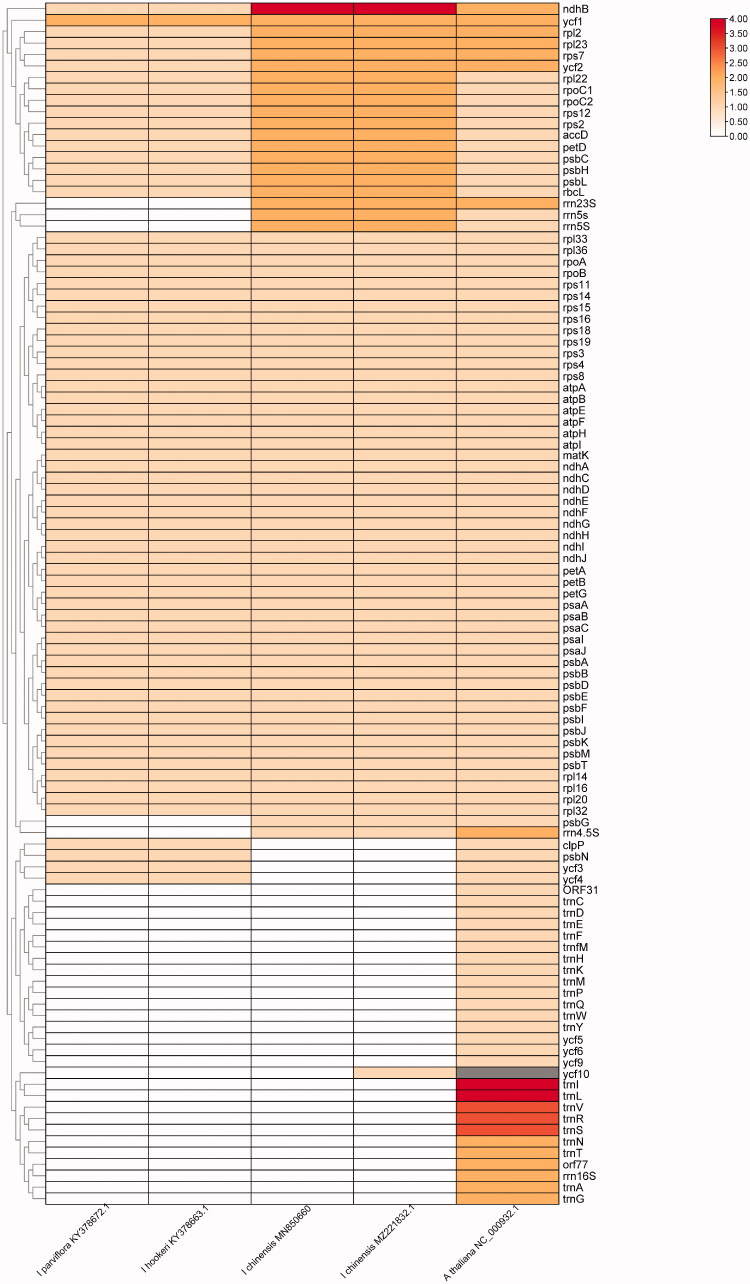
Gene deletion in *Ixora* genus.

**Figure 3.**
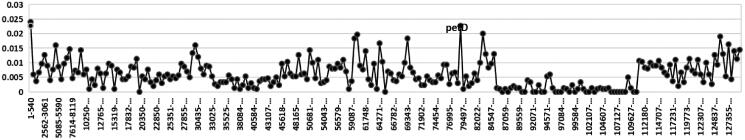
Gene polymorphism in *Ixora* genus.

**Figure 4.**
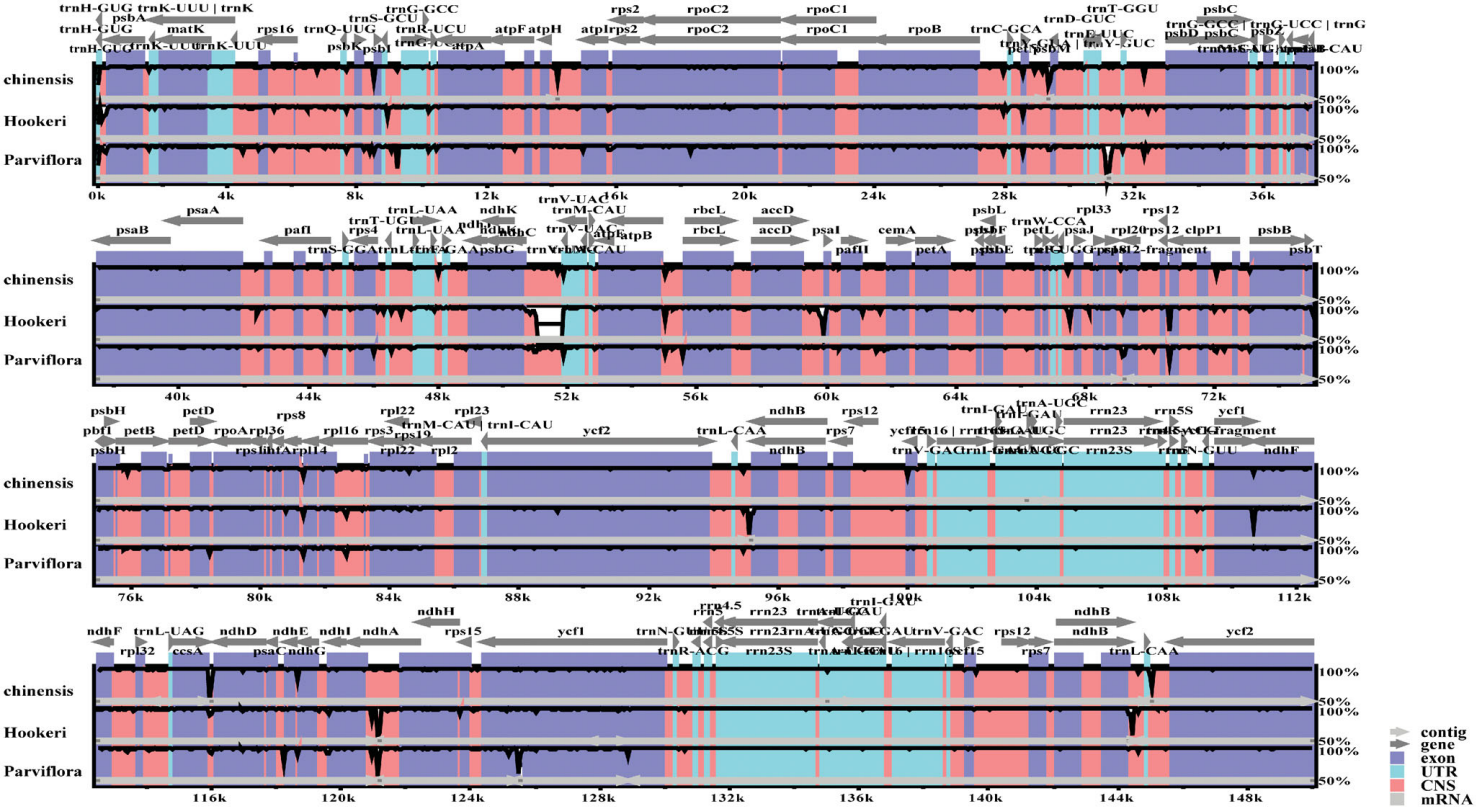
Sequence identity plot comparing four species of subfamily Ixora with *I. chinensis* (MN850660.1) as the reference annotated genome.

In order to understand the phylogenetic relationship between *I. chinensis* and other species of Ixora, the complete chloroplast sequence of 43 species was downloaded from NCBI as the phylogenetic dendrogram. The tree phylogenetic with maximum likelihood (ML) analysis method was performed using RaxML software v 8.2.9, of which the bootstrap values were calculated using 1000 replicates under the GTRGAMMAI substitution model in CIPRES (Stamatakis 2014). The reconstructed phylogeny showed that *I. chinensis* had the closest relationship with *I. chinensis* (MN850660.1) (Figure 4), which can provide a theoretical basis for further study on the phylogeny and evolution of Rubiaceae plants (Figure 5).

**Figure 5.**
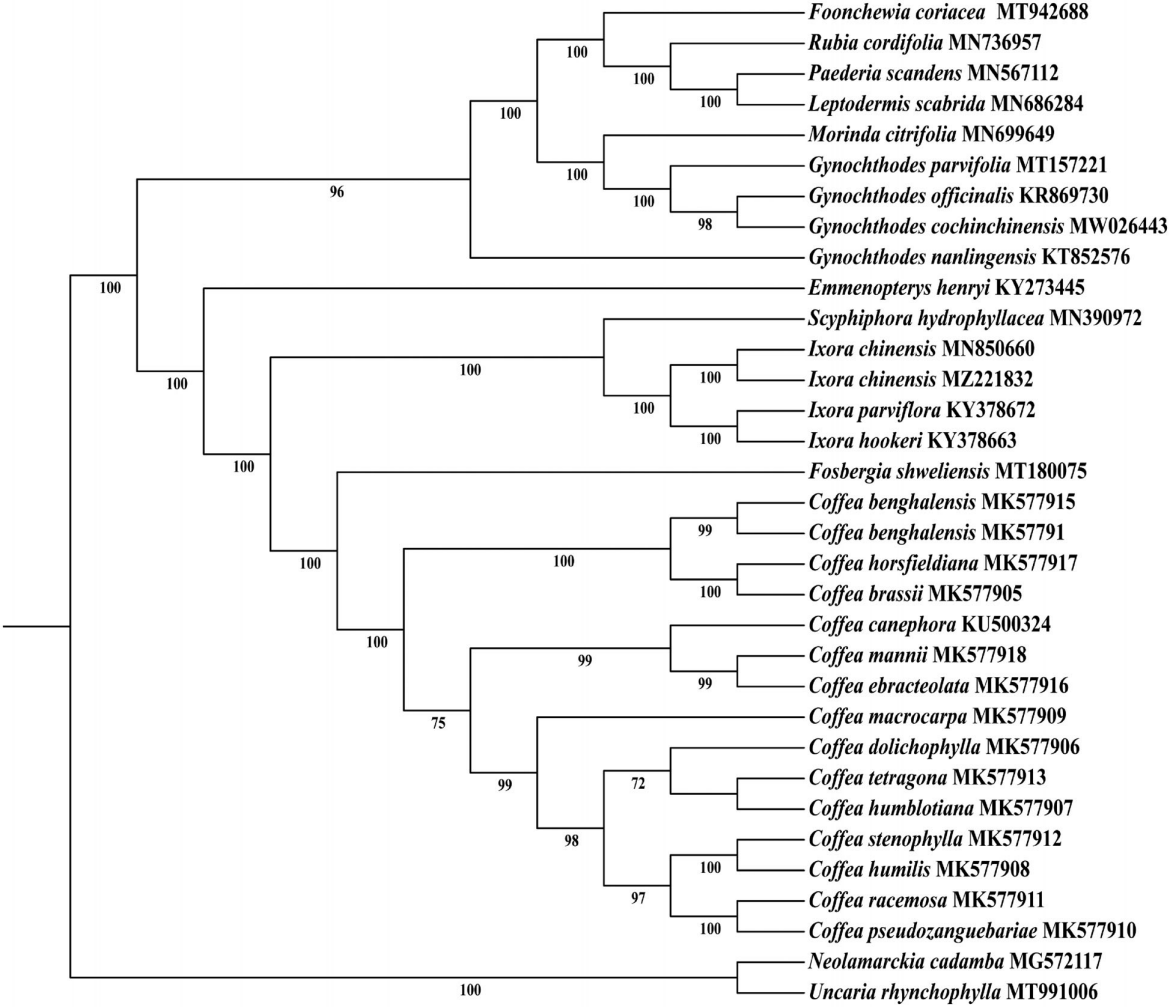
The maximum likelihood (ML) phylogenetic tree was constructed based on the complete chloroplast genomes of 34 species. The number next to each node represents the bootstrap values.

## Data Availability

The data that support the findings of this study are openly available in GenBank of NCBI at https://www.ncbi.nlm.nih.gov, reference number MZ221832. The associated BioProject, SRA, and Bio-Sample numbers and are PRJNA743157 SRR15020882 and SAMN20000888, respectively.

## References

[CIT0001] Andrews S. 2014. FastQC: q quality control tool for high throughput sequence data. http://wwwbioinformaticsbabrahamacuk/projects/fastqc/.

[CIT0002] Bankevich A, Nurk S, Antipov D, Gurevich A, Dvorkin M, Kulikov A, Lesin V, Nikolenko S, Pham S, Prjibelski A, et al. 2012. SPAdes: a new genome assembly algorithm and its applications to single-cell sequencing. J Comput Biol. 19(5):455–477. 22506599 10.1089/cmb.2012.0021 PMC3342519

[CIT0003] Bhagyasri Y, Ali P, Raja M, Reddy N, Praveen D, Mounika K, Latha D, Parameshwari N. 2019. Determination of in-vitro anti microbial activity and anti-diabetic activity of *Ixora chinensis*. AJPHR. 7(3):6–12.

[CIT0004] Chen L-Y, Chu C-Y, Huang M-C. 2003. Inflorescence and flower development in Chinese *Ixora*. JASHS. 128(1):23–28.

[CIT21652594] Kim S-M, Kuzuyama T, Chang Y-J, Kim S-U. 2006. Cloning and characterization of 2-C-methyl-D:-erythritol 2,4-cyclodiphosphate synthase (MECS) gene from Ginkgo biloba. Plant Cell Rep. 25(8):829–835. doi:10.1007/s00299-006-0136-3. 16528563 16528563

[CIT0005] Li T, Cai H, Wang T, Fu Y, Yang W, Zhao A, Cui Z, Wang J. 2019. Plant regeneration in *Ixora chinensis* from young leaves. Plant Cell Tiss Organ Cult. 139(3):605–608.

[CIT0006] Ly S, Garavito A, De Block P, Asselman P, Guyeux C, Charr J-C, Janssens S, Mouly A, Hamon P, Guyot R. 2020. Chloroplast genomes of Rubiaceae: comparative genomics and molecular phylogeny in subfamily Ixoroideae. PLoS One. 15(4):e0232295. 32353023 10.1371/journal.pone.0232295 PMC7192488

[CIT0007] Rastogi RM, Mehrotra BN. 1990. Compendium of Indian medicinal plants. Vol. 1. Lucknow: Central Drug Research Institute and Publications & Information Directorate, New Delhi; p. 388–389.

[CIT0008] Stamatakis A. 2014. RAxML version 8: a tool for phylogenetic analysis and post-analysis of large phylogenies. Bioinformatics. 30(9):1312–1313. 10.1093/bioinformatics/btu033 PMC3998144 24451623

[CIT0009] Tillich M, Lehwark P, Pellizzer T, Ulbricht-Jones E, Fischer A, Bock R, Greiner S. 2017. GeSeq - versatile and accurate annotation of organelle genomes. Nucleic Acids Res. 45(W1):W6–W11. 28486635 10.1093/nar/gkx391 PMC5570176

